# Guided and unguided neural organoids play complementary roles in studying neurodevelopment and neuroinflammation

**DOI:** 10.31744/einstein_journal/2026AO1716

**Published:** 2026-02-12

**Authors:** Raphaella Josino, Bruno Yukio Yokota-Moreno, Isabella de Sousa Nóbrega, André Luíz Teles e Silva, Melissa Bernardini Bachir Moysés, Guilherme Grecco Ferreira, Mariana Silva Branquinho, Elisa Varella Branco, Maria Rita Passos-Bueno, Andrea Laurato Sertié

**Affiliations:** 1 Hospital Israelita Albert Einstein Faculdade Israelita de Ciências da Saúde Albert Einstein São Paulo SP Brazil Faculdade Israelita de Ciências da Saúde Albert Einstein, Hospital Israelita Albert Einstein, São Paulo, SP, Brazil.; 2 Universidade de São Paulo Instituto de Biociências Departamento de Genética e Biologia Evolutiva São Paulo SP Brazil Centro de Estudos do Genoma Humano e Células-tronco, Departamento de Genética e Biologia Evolutiva, Instituto de Biociências, Universidade de São Paulo, São Paulo, SP, Brazil.

**Keywords:** Induced pluripotent stem cells, Guided dorsal forebrain neural organoids, Unguided neural organoids, Malformations of cortical development, Neuroinflammatory diseases

## Abstract

**Objective:**

This study aimed to compare guided dorsal forebrain neural organoids with unguided neural organoids, focusing on differences in structural organization, cellular composition, and functional properties.

**Methods:**

Using the same human induced pluripotent stem cell line, we applied two established differentiation protocols in parallel to generate guided and unguided neural organoids.

**Results:**

Guided neural organoids exhibited reproducible cytoarchitecture, relatively homogeneous morphology, and robust network activity, making them particularly well-suited for modeling specific aspects of cortical neurodevelopment and neurodevelopmental disorders. In contrast, unguided neural organoids displayed greater heterogeneity in morphology and cellular composition, including prominent development of astrocytes, microglia, and choroid plexus-like structures that respond to inflammatory stimuli, positioning them as valuable models for studying neuroinflammation during brain development.

**Conclusion:**

These findings emphasize the importance of selecting neural organoids protocols based on specific research questions, and suggest that the guided and unguided approaches can complement each other to provide insights into neurodevelopmental and neuroinflammatory processes.

## Highlights

■Guided cortical neural organoids show consistent cytoarchitecture and robust network activity.

■Unguided neural organoids are heterogeneous, with glia, microglia, and choroid plexus cells.

■Guided neural organoids suit cortical development and neurodevelopmental disease studies.

■Unguided neural organoids are ideal for modeling neuroinflammation and diverse cell interactions.

## In Brief

Neural organoids model human brain development using guided or unguided approaches. Josino et al. showed that guided cortical neural organoids have consistent cytoarchitecture and robust network activity, ideal for cortical development and disease studies, while unguided neural organoids are more diverse, with glia, choroid plexus-like structures, and inflammatory responses, suited for neuroinflammation research.

## Graphical abstract

**Figure:**
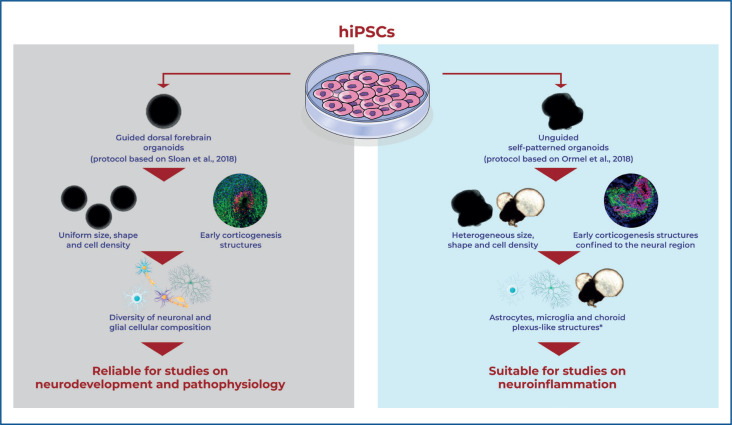

## INTRODUCTION

Investigating the biological mechanisms that drive normal human brain development and disease presents several challenges. These includes the brain's complex nature, limited access to human brain tissue, substantial variability across neurological disorders, and the limited capacity of traditional two-dimensional (2D) culture systems and animal models to accurately replicate the cellular, molecular, and structural features of human neurobiology.^(1)^ In this context, neural organoids (NOs) derived from pluripotent stem cells—particularly human induced pluripotent stem cells (hiPSCs)—have emerged as powerful tools to overcome these limitations.^(2)^ Neural organoids are three-dimensional (3D) culture systems that reproduce key aspects of brain tissue architecture and function *in vitro*. By more closely mimicking the complexity of the human brain, NOs provide a robust platform for studying neurodevelopment and the mechanisms underlying neurological diseases.^(3–5)^

Neural organoids can be generated using either guided or unguided differentiation protocols, depending on the desired level of control over cell fate.^(6,7)^ Guided protocols emply specific growth factors and signaling molecules to direct the development of defined brain regions, such as the cortex, hippocampus, or midbrain.^(8–10)^ In contrast, unguided protocols rely on the spontaneous self-organization of hiPSCs, resulting in the stochastic formation of various brain regions within a single organoid.^(11–13)^ While guided protocols offer a more homogeneous and region-specific model—useful for investigating targeted aspects of brain development and disease—unguided protocols better capture the broader cellular diversity and complexity characteristic of early brain formation.^(5)^

Both guided and unguided NOs, particularly those derived from patient-specific hiPSCs, have successfully recapitulated disease-related phenotypes associated with early embryonic disruptions in cortical cytoarchitecture, such as microcephaly, macrocephaly, and autism spectrum disorder (ASD).^(11,14,15)^ Beyond genetic conditions, NOs also serve as valuable platforms for investigating the impact of environmental risk factors on brain development. They have been used to model the effects of oxygen deprivation,^(16)^ maternal immune activation,^(17)^ and viral infections such as Zika virus.^(18,19)^ However, studying neuroinflammation in guided NOs remains challenging, as these systems often lack non-neural cell types such as microglia. This limitation reduces their ability to fully capture immune responses and their influence on neural development.

As the field of NOs continues to expand rapidly, selecting an appropriate organoid protocol depends on the specific research objectives and applications. A comprehensive understanding of how different NO protocols influence developmental trajectories and cellular composition is essential for optimizing their use in disease modeling and experimental neuroscience. Despite this, direct comparisons between guided and unguided NOs remain limited, with few studies evaluating both approaches in parallel.^(19,20)^

## OBJECTIVE

This study aimed to compare two distinct neural organoid models and evaluate their potential for investigating neurodevelopment and neuroinflammation.

## METHODS

### Generation of neural organoids

The hiPSC line used in this study was originally derived from stem cells of human exfoliated deciduous teeth (SHED) obtained from a neurotypical male child. This line has been shown to express pluripotency-associated markers and to efficiently differentiate into neural progenitor cells and neurons in 2D cultures.^(21)^

**Figure 1:**
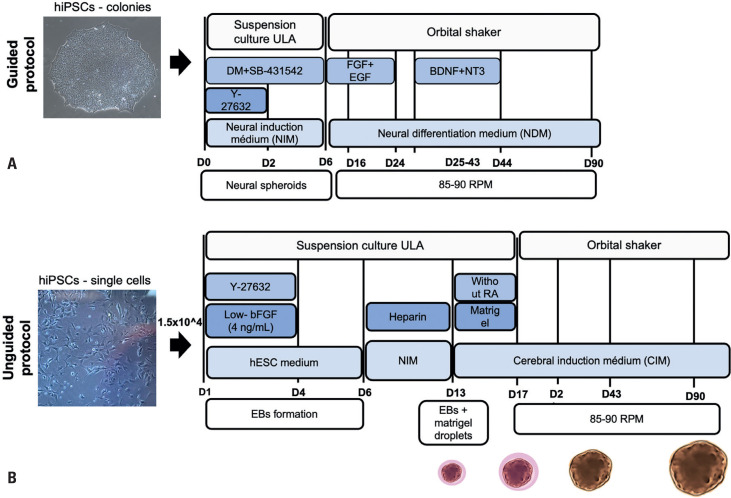
Schematic comparison of guided (Sloan protocol) and unguided (Ormel protocol) approaches for neural organoid generation. (A) Guided protocol. Key steps include: Day 0 (D0) – Transfer of intact hiPSC colonies to suspension culture in ultra-low attachment (ULA) plates containing Neural Induction Medium (NIM) supplemented with SMAD inhibitors [dorsomorphin (DM) and SB-431542] and ROCK inhibitor (Y-27632). Days 2–5 – Maintenance of neural spheroids in NIM with DM and SB. Days 6–24 – Transition to Neural Differentiation Medium (NDM) supplemented with bFGF and EGF. Days 25–43 – Culture in NDM supplemented with BDNF and NT3. From Day 44 onward – Culture in NDM without growth factors. From D6 onward, organoids are maintained on an orbital shaker (85–90 RPM). Maturation: Organoids are cultured for 30, 60, and 90 days. (B) Unguided protocol. Key steps include: Day 0 – Seeding of dissociated hiPSCs (~1.5×10^4^ cells) in hESC medium supplemented with Y-27632 and low concentrations of bFGF. Day 6 – Transfer of resulting embryoid bodies (EBs) to NIM low-dose heparin in ULA plates. Day 13 – Embedding of individual EBs in Matrigel droplets and culture in Cerebral Induction Medium (CIM) lacking retinoic acid (RA). Day 17 onward – Organoids are transferred to an orbital shaker (85–90 RPM). Maturation: Organoids are cultured in CIM for 30, 60, and 90 days. While both protocols involve long-term organoid maturation, they differ in their initial seeding strategies, induction methods, and media composition, which are designed either to enhance neuroectodermal specification (Sloan protocol) or minimize early patterning cues (Ormel protocol), thereby promoting broader cellular diversity

Cortical organoids were generated using a guided differentiation protocol adapted from Sloan et al.,^(10)^ with minor modifications (Figure 1A). This adapted protocol was optimized by our team to produce well-structured dorsal forebrain organoids from both urine- and SHED-derived hiPSCs.^(22,23)^ Briefly, hiPSCs were cultured on vitronectin-coated (0.5*μ*g/cm^2^) 60mm dishes until colonies reached approximately 2.5mm in diameter. Colonies were then detached using dispase and transferred (approximately seven per well) to ultra-low attachment 6-well plates for suspension culture in Neural Induction Medium (NIM) [DMEM/F12 (Thermo Fisher Scientific), 20% KnockOut Serum Replacement (KSR, Thermo Fisher Scientific), 1% Non-Essential Amino Acids (NEAA, Thermo Fisher Scientific), 0.5% GlutaMAX (Thermo Fisher Scientific), 1% Penicillin-Streptomycin (Thermo Fisher Scientific), and 0.1mM 2-Mercaptoethanol (Sigma Aldrich)], supplemented with the SMAD pathway inhibitors Dorsomorphin (DM, 5*μ*M, Sigma Aldrich), and SB-4321542 (SB, 10 *μ*M, Sigma Aldrich), along with the ROCK inhibitor Y-27632 (10*μ*M, Sigma Aldrich) on Day 0. From Days 2 to 5, the resulting neural spheroids were maintained in NIM supplemented with DM and SB, with daily medium changes. On Day 6, the medium was replaced with Neural Differentiation Medium (NDM) [Neurobasal A (Thermo Fisher Scientific), 2% B27 supplement without vitamin A (Thermo Fisher Scientific), 1% GlutaMAX (Thermo Fisher Scientific), 1% Penicillin-Streptomycin (Thermo Fisher Scientific)], supplemented with basic fibroblast growth factor (bFGF, 20 ng/ml, Thermo Fisher Scientific) and epidermal growth factor (EGF, 20ng/ml, Peprotech). Organoids were maintained under these conditions until Day 24, with medium changes every other day. To enhance nutrient and oxygen diffusion, an orbital shaker (85-90 RPM) was introduced starting on Day 6. From Days 25 to 43, the medium was supplemented with brain-derived neurotrophic factor (BDNF, 20 ng/ml, PeproTech) and neurotrophin-3 (NT3, 20 ng/ml, PeproTech), with medium changes every 2-3 days. From Day 44 onward, organoids were maintained in unsupplemented NDM, with medium changes every 4 days. Organoids were cultured for 30, 60, and 90 days, with samples collected at each time point for subsequent analyses.

Unguided neural organoids (NOs) were generated based on the protocol described by Ormel et al.,^(13)^ which allows for the emergence of a wider range of cell types, including microglia and choroid plexus cells. Briefly, hiPSCs at 70-80% confluency were dissociated into a single-cell suspension using Accutase (Thermo Fisher Scientific) and seeded into ultra-low attachment U-bottom 96-well plates at ~1.5×10^4^ cells per well in 150*μ*L hESC medium [DMEM/F12 (Thermo Fisher Scientific), 20% KSR (Thermo Fisher Scientific), 3% Fetal Bovine Serum (FBS, Thermo Fisher Scientific), 1% NEAA (Thermo Fisher Scientific), 1% GlutaMAX (Thermo Fisher Scientific), 1% Penicillin-Streptomycin (Thermo Fisher Scientific), 496 *μ*M 2-Mercaptoethanol (Sigma Aldrich)], supplemented with ROCK-inhibitor Y-27632 (50*μ*M) and bFGF (4ng/mL) for the first 4 days. Around Day 6, the resulting embryoid bodies (EBs) were transferred to 24-well low-attachment plates containing 500*μ*L of NIM [DMEM/F12 (Thermo Fisher Scientific), 1% N2 Supplement (Thermo Fisher Scientific), 1% GlutaMAX (Thermo Fisher Scientific), 1% NEAA (Thermo Fisher Scientific)], supplemented with heparin (0.1 *μ*g/mL) (Sigma Aldrich). On Day 13, individual EBs were embedded in 10*μ*L of Matrigel (Corning) droplets and cultured in Cerebral Induction Medium (CIM) [DMEM/F12 (Thermo Fisher Scientific): Neurobasal medium (Thermo Fisher Scientific) (1:1), 1% GlutaMAX (Thermo Fisher Scientific), 1% Penicillin-Streptomycin (Thermo Fisher Scientific), 0.5% NEAA (Thermo Fisher Scientific), 0.5% N2 supplement (Thermo Fisher Scientific), 1% B27 supplement (Thermo Fisher Scientific), 62.5 *μ*L insulin (Sigma Aldrich) and 87.5*μ*L 2-mercaptoethanol (Sigma Aldrich) diluted in DMEM (1:100)]. From Day 17 onward, Matrigel-embedded EBs were cultured on an orbital shaker at 85-90 RPM, with medium changes twice per week. Organoids were maintained in CIM for 30, 60, and 90 days, with samples collected at each time point for subsequent analyses.

Two independent rounds of organoid differentiation were performed using both protocols. All experiments were conducted separately on organoids from Batch 1 and Batch 2, both of which yielded consistent results. The data presented are from one representative batch of differentiation that accurately reflects the consistent outcomes observed across both batches.

### Measurements of neural organoid size

The overall morphology of both guided and unguided organoids was evaluated using bright-field imaging with the EVOS M5000 Imaging System. To quantify the size and growth over time, surface area measurements were performed using ImageJ macro toolsets (National Institutes of Health, USA; http://imagej.nih.gov/ij). For unguided NOs, measurements included the entire organoid structure, encompassing the prominent choroid plexus-like regions. To specifically evaluate the effects of interleukin-17A (IL-17A) treatment on choroid plexus-like structures, the diameters of these regions were measured separately in both IL-17A-treated and vehicle-treated organoids at Day 45. Size measurements were obtained from n=20-150 organoids per time point for growth analysis, and from n=6-7 organoids per group for IL-17A treatment comparisons.

### Immunocytochemistry staining and image quantification

Immunocytochemistry was performed on sectioned organoid samples to identify and localize neural regions and characterize cellular composition. Initial staining at Day 60 of differentiation was conducted to assess the presence and spatial distribution of neural regions in both guided and unguided organoids (n=2-3 organoids per type). To further investigate developmental trajectories, additional immunostaining analyses were performed at Days 30, 60, and 90 on sectioned guided organoids and the neural regions within unguided organoids (n=2-3 organoids per time point). The protocol followed the method described by Teles e Silva et al.^(22)^ Briefly, whole organoids were fixed in 4% paraformaldehyde in PBS at 4°C, and then submerged in 30% sucrose solution until they sank. Subsequently, organoids were transferred to cryomolds, embedded in OCT compound, and stored at −80°C until sectioning. Frozen organoids were sectioned at 15 *μ*m thickness using a cryostat and mounted onto glass slides. For immunostaining, organoid sections were permeabilized and blocked in a solution containing 5% donkey serum and 1% Triton X-100 in PBS for 1 h at room temperature. Primary antibody incubation was performed overnight at 4°C, followed by PBS washes. Secondary antibodies conjugated to Alexa Fluor 594 or Alexa Fluor 488 were applied for 1 h at room temperature, and nuclei were counterstained with DAPI. Fluorescence images were acquired using a Zeiss LSM 710 confocal microscope. For image quantification, marker expression was measured in ImageJ within defined areas ("bins," 10*μ*m² each) per organoid section. For each time point, 2-4 bins were analyzed per section from 2-3 organoids, totaling 4-12 bins per time point. To account for variations in cell density, all measurements were normalized to the total number of DAPI-stained nuclei within the corresponding quantified bins. A detailed list of primary antibodies utilized is provided in Table 1S, Supplementary Material.

### Western blot analyses

Western blot analysis was conducted as described in Teles e Silva et al.,^(22)^ using protein extracts from whole organoid samples collected at differentiation Days 30, 60, and 90 (n=3 organoids per time point). Briefly, organoids were lysed in RIPA buffer to extract total proteins, followed by sonication to ensure complete homogenization. The protein concentration of each lysate was quantified using the Pierce BCA protein assay, and absorbance was measured with the Glomax^®^ Discover Microplate Reader. Protein samples (20*μ*g) were resolved by SDS-PAGE on 8-10% polyacrylamide gels and transferred onto nitrocellulose membranes for immunoblotting. Membranes were blocked with 5% non-fat milk or BSA in TBST (Tris-buffered saline with Tween-20) and incubated overnight at 4°C with primary antibodies (Table 1S, Supplementary Material). After washing, membranes were incubated for 1 h at room temperature with horseradish peroxidase-conjugated secondary antibodies (anti-rabbit or anti-mouse). Protein bands were detected using an ECL substrate and visualized with the ChemiDoc MP Imaging System.

### Treatment with IL-17A

Recombinant human IL-17A protein (PHC9171, Thermo Fisher Scientific) was used at a concentration of 50ng/mL^(24)^ to assess the inflammatory effects of IL-17A on unguided organoids. Cultures were treated with either vehicle (ultrapure water) or IL-17A from Days 25 to 43. As described above, bright-field images were collected on Day 45 to evaluate the morphological changes, with a particular focus on alterations in the choroid plexus-like structures.

### Multielectrode array

Spontaneous extracellular field potentials were recorded from intact guided and unguided organoids on Days 30, 40, 50, and 60 of differentiation (n=3 per group), using a 24-well CytoView multielectrode array (MEA) plate (Axion Biosystems), with each well containing 16 electrodes arranged in a 4 × 4 grid. Organoids were plated on Day 30 and maintained in the same wells through Day 60. During the first week, they were cultured in their respective media; thereafter, BrainPhys medium (Stemcell Technologies) was used for all groups. Recordings were performed using the Maestro Edge system and AxIS software (version 274 1.0, Axion Biosystems), applying a bandwidth filter from 10 Hz to 4.5 kHz. Spike detection was performed using an adaptive threshold set at 4.5 times the standard deviation of the estimated noise for each electrode. Prior to recording, plates were left undisturbed in the Maestro instrument for 5 min, followed by a 10-min recording session. Data were analyzed to determine the mean firing rate (Hz).

### Statistical analysis

Results are represented as mean ± SEM. Statistical analyses were performed using GraphPad Prism version 8. One-way ANOVA was used to compare multiple groups and assess overall statistical significance. For pairwise comparisons between groups, particularly when data did not meet the assumptions of parametric tests, the Mann-Whitney U test was applied. A p<0.05 was considered statistically significant.

### Disclosures

The study was conducted following the Declaration of Helsinki and approved by the Institutional Review Board and Ethics Committee of *Hospital Israelita Albert Einstein*. Written informed consent was obtained from each participant and their parents/legal guardians.

## Results

### Guided and unguided neural organoids exhibit distinct macroscopic morphologies

To systematically compare NOs generated by guided and unguided approaches, we used the same hiPSC line and applied two established protocols in parallel. The guided protocol, described by Sloan et al.,^(10)^ employs SMAD inhibition and specific growth factors to actively direct differentiation toward dorsal cortical structures of the neuroectodermal lineage. In contrast, the unguided protocol, described by Ormel et al.,^(13)^ relies on a more spontaneous differentiation approach, producing a less directed and more diverse cellular composition, including cell types derived from all three germ layers. Notably, this unguided approach has been shown to improve the generation of microglia (mesodermal origin) and other non-neural cell types, such as choroid plexus cells.

**Figure 2:**
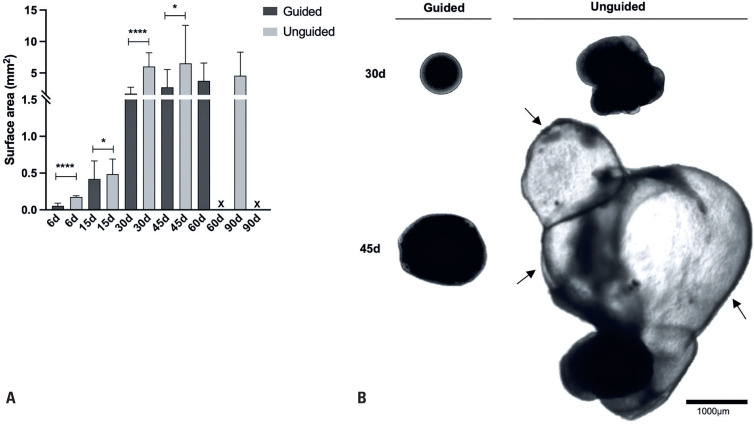
Comparison of macroscopic morphology and size of neural organoids generated using the guided and unguided protocols throughout culture. (A) Graph comparing the size of guided and unguided NOs, based on the average 2D surface area (mm^2^) at different time points (Days 6, 15, 30, 45, 60, and 90). Data are presented as mean ± SEM (n=20–150 organoids per time point). By Day 60, quantitative analysis of unguided NO size became no longer feasible. (B) Representative bright-field images of guided and unguided NOs at Days 30 and 45. Scale bars, 1000 *μ*m. Arrows indicate choroid plexus-like structures visible in the unguided NOs

First, we analyzed the growth dynamics of NOs generated using these protocols at different time points: Days 6, 15, 30, 45, 60, and 90 (Figure 2A). By Day 6, the unguided NOs were significantly larger than those produced with the guided protocol, indicating faster growth during the early stages of development. This size difference remained significant at Day 15 and became even more pronounced by Days 30 and 45. At these later time points, the unguided NOs began to exhibit prominent choroid plexus-like structures, visible as fluid-filled, bubble-like formations, consistent with previous observations by Kong et al.^(25)^ using the same protocol (Figure 2B). After Day 60, accurate size quantification of the unguided NOs was no longer feasible, as their dimensions exceeded the microscope's field of view. Conversely, the guided NOs remained within the view throughout the time course and showed a steady increase in size, reaching an average 2D surface area of approximately 4mm² by Day 60. Beyond this point, their growth appeared to plateau.

*p<0.05; ****p<0.0001.

### Guided and unguided neural organoids exhibit significant differences in cell composition

**Figure 3:**
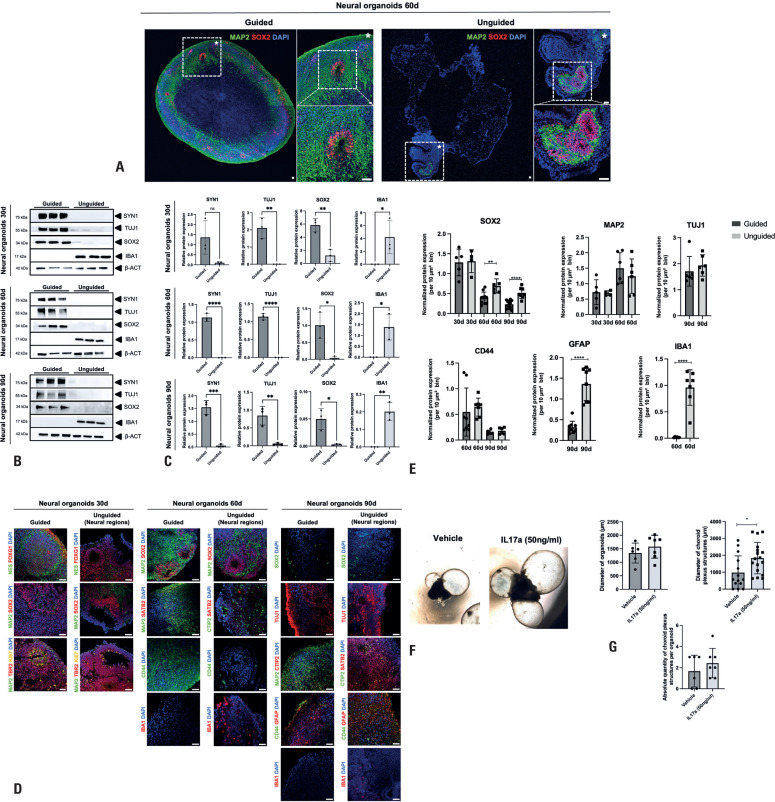
Characterization of neural organoids through 2D immunocytochemistry and Western blotting. (A) Representative immunostaining of guided and unguided NOs at Day 60. Guided NOs (left) exhibit VZ-like structures composed of neural progenitor cells (SOX2+) surrounded by neurons (MAP2+) distributed throughout the entire structure, whereas the unguided NOs (right) develop these structures in specific, confined neural regions. Detached areas show magnified views for clearer visualization. Nuclei are stained with DAPI. Scale bar, 50um. (B) Representative immunoblot images showing the expression patterns of neural progenitor (SOX2), neuronal (SYN-1, TUJ-1), and microglia (IBA1) markers in lysates from whole guided and unguided NOs at Days 30, 60, and 90 (n=3 organoids per time point). β-actin was used as the loading control. (C) Quantification graphs of TUJ1, SYN1, SOX2, and IBA1 relative expression levels. Data are shown as mean ± SEM (n=3 organoids per time point). Protein levels were normalized by β-actin levels. (D) Representative immunostaining of guided NOs and neural regions of unguided NOs cultured for 30, 60, and 90 days. At Day 30, both models exhibited robust VZ-like structures positive for neural progenitor markers [NESTIN (NES), SOX2, FOXG1] and intermediate progenitor markers (TBR2). These SOX2-positive VZ-like structures persisted in 60-Day NOs. By Days 60 and 90, both guided and unguided NOs showed the presence of lower-layer cortical neurons (CTIP2), upper-layer cortical neurons (SATB2), and astrocytes (CD44 and GFAP). Notably, IBA1-positive microglia were observed exclusively in unguided NOs. (E) Quantification of the relative expression of neural progenitor (SOX2), neuronal (MAP2, TUJ1), astrocytic (CD44, GFAP), and microglial (IBA1) markers was performed on 4–12 bins (areas of 10 *μ*m² each) per organoid per time point. (F) Representative bright-field images showing unguided NOs at Day 45 treated with either vehicle or IL-17A. (G) Quantification graphs showing the following measurements for unguided NOs at Day 45 following vehicle or IL-17A treatment: average diameter (*μ*m), number of choroid plexus-like structures, and diameter (*μ*m) of the choroid plexus-like structures. Data are presented as mean ± SEM (n=6–7 organoids per treatment)

Given the presence of prominent choroid plexus-like structures in the unguided NOs during bright-field examination, we sought to identify and localize the neural regions within these organoids. To this end, immunocytochemistry was performed on cryosectioned NOs at Day 60 to compare guided and unguided samples. Sections were stained for neural progenitors (SOX2) and neuronal (MAP2) markers to assess the presence and distribution of neural populations. Our analysis revealed that guided NOs formed neural rosettes—prospective ventricular-zone-like (VZ-like) structures—composed of SOX2+ neural progenitor cells surrounded by MAP2+ neurons, distributed throughout the organoid. In contrast, the unguided NOs exhibited a dominant presence of choroid plexus-like regions, with SOX2+/MAP2+ rosettes restricted to smaller, localized regions within the organoid (Figure 3A). Moreover, the size of these neural regions varied among the unguided NOs, reflecting greater heterogeneity in their neural tissue organization.

*p<0.05; **p<0.01; ****p<0.0001.

To quantitatively assess neural content, we performed Western blot analyses on protein lysates from whole guided and unguided NOs—including both neural and non-neural regions in the latter—at Days 30, 60, and 90. As expected, guided NOs exhibited significantly higher expression of neural progenitor (SOX2) and neuronal (SYN-1, TUJ-1) markers across all time points, confirming the comparatively reduced neural content in the unguided NOs. Furthermore, these markers showed consistent and reproducible expression patterns in the guided samples, emphasizing the robustness of the guided differentiation protocol. Conversely, the microglial marker IBA1 was detected exclusively in unguided NOs, indicating the absence of microglia in the guided model (Figures 3B and C).

To further characterize the developmental trajectories and cellular composition of both NO types, we extended the immunocytochemistry analyses to Days 30, 60, and 90, staining for key markers of neural progenitors, neurons, and glial cells across time points. For the unguided NOs, only neural regions were analyzed, with choroid plexus-like structures deliberately excluded to enable a focused and comparable assessment of neurodevelopmental features. Qualitative assessment revealed that both guided NOs and the neural regions of unguided NOs recapitulated fundamental features of human cortical development. By Day 30, both models had developed VZ-like structures composed of proliferating neural progenitor cells (SOX2+, FOXG1+, Ki67+), surrounded by intermediate progenitors (TBR2+) and immature neurons (MAP2+). By Days 60 and 90, both NO types contained neurons characteristic of lower (CTIP2+) and upper (SATB2+) cortical layers, along with astrocytes (CD44+, GFAP+). Consistent with the Western blot findings, microglia (IBA1^+^) were found exclusively in the unguided NOs (Figure 3D). Quantitative analysis of selected markers revealed a modest increase in SOX2+ neural progenitors within the neural regions of the unguided NOs at Days 60 and 90, and a significant increase in GFPA+ astrocytes by Day 90. This analysis also reaffirmed the presence of microglia in the unguided NOs, highlighting the marked differences in cellular composition between the two models (Figure 3E).

Finally, considering that the choroid plexus is known to respond to inflammatory stimuli *in vivo,*^(26,27)^ and given the prominent expansion of choroid plexus-like structures in the unguided NOs, we sought to evaluate their responsiveness to IL-17A, a pro-inflammatory cytokine linked to maternal immune activation and ASD.^(28)^ Treatment with IL-17A did not affect the diameter of the neural region or the overall number of choroid plexus-like structures; however, it resulted in a significant increase in the size of these structures (Figures 3F and G). This observation suggests that the choroid plexus-like structures within the unguided NOs are capable of responding to inflammatory signals.

Taken together, these results suggest that both guided and unguided NOs are valuable models for studying the early stages of human corticogenesis. However, the neural regions within unguided NOs were significantly smaller than those in guided NOs, which may limit their use in studies requiring larger, more reproducible, and expansive neural areas. In contrast, the unguided NOs, generated using the protocol described by Ormel et al,^(13)^ uniquely support the development of immune components, including astrocytes, microglia, and choroid plexus-like structures, making them particularly suitable for studying neuroinflammation.

### Microelectrode array analysis reveals significant differences in network activity between guided and unguided neural organoids

**Figure 4:**
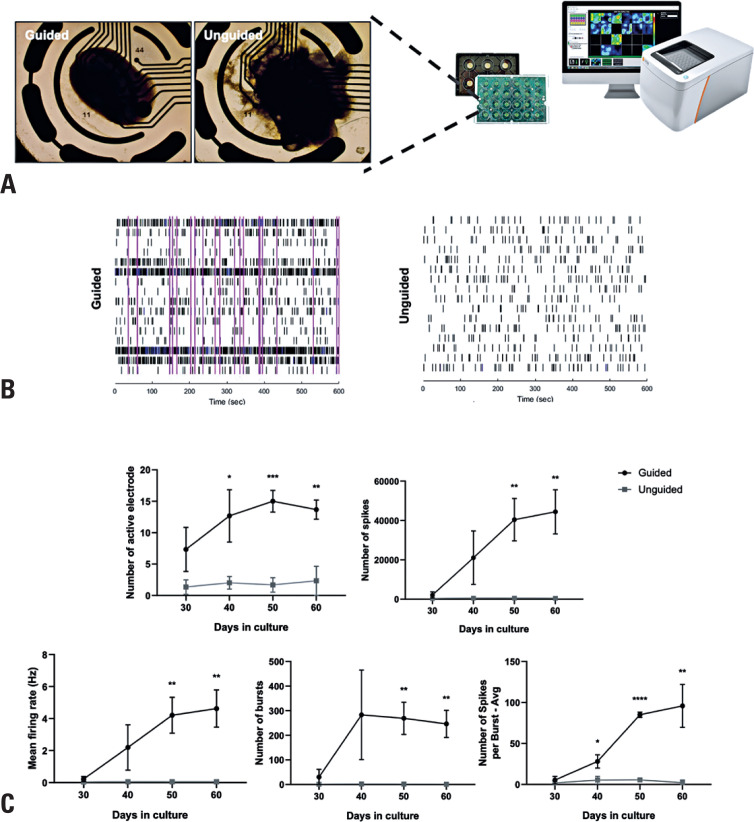
Microelectrode array analysis of guided and unguided neural organoids. (A) Schematic representation of electrophysiological recordings from intact NOs using the Maestro system. (B) Representative raster plots depicting spontaneous neuronal firing patterns recorded from guided and unguided NOs at Day 30 of differentiation. (C) Quantitative analysis of MEA parameters, including the average number of active electrodes, total spike count, mean firing rate (Hz), number of bursts, and spikes per burst across multiple time points (Days 30, 40, 50, and 60). Data are presented as mean ± SEM (n=3 organoids per group)

To evaluate the functional maturation of NOs generated using the guided and unguided protocols, we monitored spontaneous extracellular activity over time using a 16-channel MEA platform (Figure 4A). Key parameters—including the number of active electrodes, total spike count, mean firing rate (Hz), number of bursts, and spikes per burst—were analyzed. Across all measures, unguided NOs exhibited significantly reduced intrinsic neuronal network activity compared to guided cortical NOs (Figure 4B and C). These functional differences align with our earlier observations, which showed that unguided NOs displayed smaller and more localized neural regions, accompanied by an increased abundance of astrocytes, microglia, and choroid plexus-like structures.

*p<0.05; **p<0.01; ***p<0.001.

## DISCUSSION

In this study, we compared guided (dorsal forebrain) and unguided NOs generated by two well-established and relatively simple protocols^(10,13)^ to facilitate more informed decisions regarding their applications. Both protocols were applied in parallel, starting from the same batch of SHED-derived hiPSCs, ensuring consistency in the starting material and eliminating variability due to genetic background.

Morphologically, the guided NOs exhibited more uniform growth patterns compared to their unguided counterparts, which showed significantly greater heterogeneity. This variability in the unguided NOs was primarily attributed to the uncontrolled expansion of choroid plexus-like structures,^(13,25)^ which often dominated the organoids and contributed to inconsistencies in size, shape, and overall organization. The presence of heparin in the unguided culture medium, known to inhibit ectodermal lineage commitment and promote the emergence of non-neural cell types,^(29)^ likely contributed to this outcome. While neural regions in the unguided NOs formed organized VZ-like structures, critical for modeling early cortical development, these regions were generally smaller and less reproducible than those in the guided NOs. This lack of uniformity highlights the challenges associated with achieving reproducible proportions of neural cells in unguided models, as they often exhibit heterogeneous tissue composition.^(6,30)^

Consistent with these morphological findings, protein analyses of whole organoid lysates revealed that guided NOs consistently expressed higher levels of key neural progenitor and neuronal maturation markers throughout the culture period. This finding is further supported by MEA recordings, which showed greater electrophysiological activity in guided NOs, reflecting their relatively higher proportion of neurons and more developed functional synaptic networks. These characteristics make guided NOs particularly well-suited for studying early cortical development and neurological disorders that primarily affect neuronal populations, such as epilepsy, ASD, and other psychiatric conditions.^(3,4,15)^ Notably, the study by Jourdon et al.,^(15)^ which employed a protocol similar to ours to generate guided forebrain NOs from hiPSCs derived from patients with idiopathic ASD, revealed consistent disruptions in neural progenitor biology and altered patterns of cortical neuron specification within the organoids.

While the absence of immune cells is a limitation of guided NOs, we observed that the neural regions of unguided NOs contained a notably higher number of astrocytes and a prominent population of microglia, key mediators of immune responses in the central nervous system that play critical roles in brain development and homeostasis.^(31,32)^ Interestingly, a recent study using the same unguided NO protocol found that microglia, rather than neurons, were the main targets of HIV infection, and that infected microglia created an inflamed and toxic microenvironment, ultimately leading to neuronal dysfunction and cell death.^(25)^ Additionally, Wenzel et al^(33)^ demonstrated that NOs with endogenously developed microglia exhibited enhanced responses to β-amyloid toxicity compared to organoids in which microglia were added via co-culture, suggesting that unguided NOs with endogenous microglia may provide a more accurate model for studying Alzheimer's disease-related pathology. Beyond microglia, the presence of choroid plexus-like structures in unguided NOs offers additional modeling opportunities. The choroid plexus plays a central role in cerebrospinal fluid (CSF) production, and the formation of the blood-CSF barrier.^(34)^ Dysfunction of the choroid plexus has been implicated in the pathogenesis of various neurological disorders, including schizophrenia, Alzheimer's disease, and multiple sclerosis.^(35–38)^ Notably, in our study, treatment of unguided NOs with the pro-inflammatory cytokine IL-17A resulted in a significant enlargement of the choroid plexus-like structures, a hallmark of ongoing neuroinflammation.^(39)^ This finding suggests that these NOs are responsive to inflammatory signals, further supporting their potential utility for modeling the interplay between immune activation and brain tissue responses.

Taken together, our findings illustrate the complementary strengths of these models: guided dorsal forebrain NOs provide detailed insights into early cortical development, neuronal differentiation, and network function, whereas unguided NOs offer a more comprehensive system to explore neuroglial interactions, particularly in the context of neuroinflammatory conditions.^(40,41)^ These results provide a valuable resource for the field and can help determine the most suitable model based on specific research objectives.

As a future direction, it would be particularly interesting to compare the findings of this study with more recently developed semi-guided organoid models,^(40)^ which balance directed differentiation with spontaneous tissue organization, aiming to optimize cellular diversity while retaining regional specificity. Moreover, multi-omic approaches—including transcriptomics, proteomics, and epigenomic profiling—could offer deeper insight into the developmental trajectories and functional states of different organoid types.^(41–43)^ Such comprehensive analyses will be instrumental in refining organoid technologies for both basic neuroscience research and translational applications in disease modeling and therapeutic development.

## CONCLUSION

The results of this study support the distinct yet complementary strengths of neural organoids generated through guided and unguided differentiation protocols. Guided forebrain neural organoids exhibit consistent structural organization, robust neuronal populations, and functional maturation, making them particularly suited for studying cortical development and neurodevelopmental disorders. In contrast, unguided neural organoids demonstrate greater cellular heterogeneity, including prominent astroglial, microglial, and choroid plexus-like components, positioning them as valuable tools for exploring neuroinflammation and neuroimmune interactions. Together, these models offer complementary platforms that can be strategically selected or combined based on specific research objectives.

## Data Availability

The data supporting the findings of this study are available from the authors upon reasonable request.
