# Supplementary material for: Guided and unguided neural organoids play complementary roles in studying neurodevelopment and neuroinflammation
Source: Einstein (Sao Paulo). 2026 Feb 12;24:eAO1716. doi: 10.31744/einstein_journal/2026AO1716 (PMC12977266; doi:10.31744/einstein_journal/2026AO1716)
Supplement: Supplementary Material [file 2317-6385-eins-24-eAO1716-suppl01.pdf]

SUPPLEMENTARY MATERIAL

Guided and unguided neural organoids play complementary roles in studying neurodevelopment and neuroinflammation

Raphaella Josino, Bruno Yukio Yokota-Moreno, Isabella de Sousa Nóbrega, André Luíz Teles e Silva, Melissa Bernardini Bachir Moysés, Guilherme Grecco Ferreira, Mariana Silva Branquinho, Elisa Varella Branco, Maria Rita Passos-Bueno, Andrea Laurato Sertié

DOI: 10.31744/einstein_journal/2026AO1716

Table 1S. Antibodies used in this study

Antigen	Company	Cat. No.	Marker of
SOX2	Millipore	AB5603	Neural progenitors
FOXG1	Abcam	ab18259	Neural progenitors
NESTIN	Millipore	MAB5326	Neural progenitors
MAP2	Millipore	MAB3418	Neurons
TUJ1	Sigma	T2200	Neurons
CTIP2	Abcam	ab18465	Lower cortical layer neurons
SATB2	Abcam	ab34735	Upper cortical layer neurons
GFAP	Millipore	AB5804	Astrocytes
CD44	Abcam	ab6124	Astrocytes
IBA1	Abcam	ab178846	Microglia
Ki67	Abcam	ab254123	Cell proliferation
Alexa Fluor 594	Thermo Fisher Scientific	A11012	Secondary antibody
Alexa Fluor 488	Thermo Fisher Scientific	A32723	Secondary antibody
Alexa Fluor 546	Thermo Fisher Scientific	A11040	Secondary antibody
Alexa Fluor 680	Thermo Fisher Scientific	A21096	Secondary antibody
